# Diagnosis and Treatment of Thoracic Outlet Syndrome in an Elderly Male Patient: A Case Report and Protocol Evaluation

**DOI:** 10.7759/cureus.79306

**Published:** 2025-02-19

**Authors:** Joshua L Dale, Harpreet Sood MD

**Affiliations:** 1 Osteopathic Medicine, William Carey University College of Osteopathic Medicine, Hattiesburg, USA; 2 Family Medicine, Covington County Hospital, Collins, USA

**Keywords:** arterial thoracic outlet syndrome, clinical orthopedics, functional positional release, manual medicine, osteopathic technique

## Abstract

Thoracic outlet syndrome (TOS) is a relatively common condition in the United States. It is characterized by numbness or pain radiating down the ulnar aspect of the affected arm. This case report discusses a 76-year-old man who presented to a family medicine clinic with symptoms that caused him significant discomfort. He was seeking medications to alleviate his pain. After osteopathic manipulative treatment (OMT), the patient's symptoms were resolved.

The osteopathic techniques utilized included myofascial release, thoracic inlet release (a lymphatic technique), and facilitated positional release. Although the patient reported improved symptomology post-treatment, he was lost to follow-up. This case suggests a potentially positive intervention using OMT for the treatment of a possible case of myofascial-induced TOS.

## Introduction

Thoracic outlet syndrome (TOS) has an estimated incidence of three to 80 cases per 1,000 individuals in adolescents and middle-aged adults [1], reflecting significant variability in diagnostic criteria and reporting standards. The actual prevalence may be higher [2] as many cases are likely underdiagnosed due to overlapping symptoms with other conditions. TOS is characterized by upper extremity pain, which may follow a shooting or tingling pattern [3]. While symptoms commonly involve the ulnar aspect of the arm and grip weakness, it can also present with more generalized pain, tingling, or a pattern resembling part of the median nerve distribution, depending on the location of compression and associated tensions [3]. Patients may also experience hand muscle atrophy or swelling, depending on the etiology.

TOS is generally classified into two categories: neurogenic and vascular [1]. Neurogenic TOS can lead to muscle atrophy due to prolonged nerve compression, while vascular TOS may cause swelling resulting from impaired blood flow or venous congestion. Although these symptoms may be present, a clinical diagnosis is typically conducted using specialized tests such as Adson's test, Roos test, and upper limb tension test [4]. These tests vary in their sensitivity and specificity. Adson's test is often criticized for its low diagnostic accuracy, while the Roos test is noted for its ability to reproduce symptoms. Incorporating these metrics can help clinicians select the most appropriate test based on the patient's presentation. While neurogenic TOS can be diagnosed using these special orthopedic tests, vascular TOS may require imaging studies such as Doppler ultrasound, angiography, or other vascular imaging techniques for accurate diagnosis [5].

Cubital tunnel syndrome, a common differential diagnosis, can be distinguished from TOS through a positive Tinel test at the elbow [3]. Misdiagnoses are possible, as conditions like cubital tunnel syndrome, cervical radiculopathy, and carpal tunnel syndrome are often mistaken for TOS [1,2,4,6]. In the U.S., physical therapy is typically the primary treatment for TOS. However, osteopathic techniques have demonstrated effectiveness in rapidly relieving symptoms. Richard Dobrusin, Doctor of Osteopathy (D.O.), reported successful treatment of TOS using first rib counter strain techniques, which involves side-bending the patient toward the dysfunctional rib and slightly rotating them away for 90 seconds [7]. If the counterstrain fails, proficiency in additional techniques, such as thrust techniques, can be valuable for physicians [7]. Although the thrust techniques may not require advanced training, some patients may be uncomfortable with them or struggle to relax enough for counterstrain. Being skilled in multiple approaches allows for more personalized care.

This case study presents an elderly man engaged in repetitive work than caused him to raise his arms over his head. He experienced symptomatic improvement following osteopathic treatment. Unfortunately, the patient was lost to follow-up, preventing an assessment of the long-term efficacy of the protocol.

## Case presentation

Clinical findings and diagnostic assessment of a 76-year-old male patient who presented to the family medicine clinic revealed shooting pain radiating down the ulnar aspect of the left arm into the fifth and fifth digits. The patient also reported progressively worsening grip strength over the past year. His occupational history included work as a long-haul truck driver and power line mechanic, involving significant activities that needed him to raise his arms over his head.

Medical history 

The patient's history was notable for hypertension, which was well-controlled with losartan. He denied any recent trauma, fever, or systemic symptoms. He also denied smoking and reported only occasional social alcohol use. His surgical history was unremarkable, and he was not taking anticoagulants or other medications that could complicate musculoskeletal treatment. A review of systems revealed occasional neck stiffness but no other musculoskeletal complaints.

Findings of physical exam

On physical examination, the patient presented with the following vitals: blood pressure 144/66 mmHg, heart rate 85 bpm, respiratory rate 12 breaths per minute, and oxygen saturation 99% on room air. The patient's radial pulses were both 2+ and equal bilaterally, and there was no skin discoloration on either of the upper extremities. The patient had a BMI of 34.5. Neurologic examination revealed reflexes that were 2+ and symmetric in the biceps, triceps, and brachioradialis bilaterally. Strength testing demonstrated 4/5 strength in the left-hand grip. All other strength tests in the upper extremity were 5/5, including bilateral upper extremities, wrist flexors, extensors, and shoulder abductors. The sensation was diminished within the ulnar distribution of the left forearm and hand. No other dermatomes demonstrated decreased sensation. The orthopedic tests conducted in this encounter were Roos Test, Phalen's test, Tinel's test at the elbow, and Spurling's Maneuver as well as an assessment of the first rib. Out of them, the Roos test was positive within seven seconds, which required the patient to lower their left arm. The first rib was depressed on the patient's left side. All other special tests conducted were found to be negative on examination. The findings and descriptions are summarized in Table 1. 

**Table 1:** Orthopedic tests conducted and their results

Orthopedic test	Findings
Roos test	Positive: Present at seven seconds
Phalen's test	Negative: No symptoms elicited
Tinel's test at the elbow	Negative: No symptoms elicited
Spurling's maneuver	Negative: No symptoms elicited
First rib assessment	Depressed first rib on the left side

Imaging and labs 

There were no other imaging or diagnostic studies that were performed during this visit. Resources such as X-ray and ultrasound would have required transfer to the hospital associated with the facility. The clinical diagnosis of TOS was based on the patient’s presentation and physical exam findings as well as the results of the special tests. 

Therapeutic intervention 

The therapeutic intervention utilized included the following osteopathic manipulative techniques, performed after obtaining informed consent: thoracic inlet myofascial release, thoracic inlet release with the arm as a lever, and facilitated positional release. For the thoracic inlet myofascial release, the patient was positioned supine while palpation identified the greatest restriction across three planes of motion. Gentle myofascial release techniques were applied, incorporating respiratory mechanics to promote tissue relaxation. In the thoracic inlet release with the arm as a lever, the patient’s arm was used as a lever to address dysfunction in the left thoracic inlet. After three minutes of sustained technique, the scalene muscles relaxed, and the first rib was successfully repositioned. For the facilitated positional release, the patient remained supine, and the left arm was elevated with the elbow flexed. Three cycles of facilitated positional release were performed, targeting residual dysfunction in the anterior and middle scalene muscles. The techniques are summarized in Table 2.

**Table 2:** Summary of the techniques utilized [8]

Technique	Description
Thoracic inlet myofascial release	A technique that can be conducted either directly or indirectly. It identifies the three planes of motion utilizing both palpation and respiratory mechanics
Thoracic inlet release with the arm as a lever	A lymphatic drainage technique in which the patient is supine and their affected side has their arm utilized as a lever. Addressing dysfunctions within the inlet, relaxing the scalenes, and releasing the first rib.
Facilitated positional release	A technique in which we elevated and flexed the patient's arm. We normally apply cycles of treatment to release the tension within the patient's scalenes

Exam findings 

The following findings were demonstrated post treatment. The patient's former positive Roos test was now negative immediately after treatment was completed, and the patient was able to maintain the arm in an elevated state for more than three minutes without symptoms. His left first rib was no longer depressed and aligned with the first rib on the right, and both hands now exhibited comparable grip strength. A summary of post-treatment results are given in Table 3.

**Table 3:** Results of the orthopedic special tests after treatment

Orthopedic test	Findings
Roos test	Remained negative after three minutes
Phalen's test	Negative: No symptoms elicited
Tinel's test at the elbow	Negative: No symptoms elicited
Spurling's maneuver	Negative: No symptoms elicited
First rib assessment	Left rib now equal to right in location

The patient also expressed resolution of the paresthesia within the ulnar aspect of the left forearm and hand. He was advised to refrain from activities that involving lifting his arm over his head for the next two weeks if possible and to rest the upper extremity. Unfortunately, the patient was lost to follow-up after this visit, preventing the assessment of long-term treatment efficacy or the need for additional interventions.

## Discussion

Osteopathy is an underutilized yet valuable approach for TOS. When applied correctly, osteopathic techniques can be effective and serve as a useful adjunct to physical therapy in clinical settings. The accurate application of special tests and proper physical exams are critical for identifying the source of entrapment and determining whether a vascular or neurological component is involved, which remains the primary challenge in diagnosing TOS.

Dobrusin's prior work demonstrated success with a counterstrain approach. However, he noted limitations when counterstrain failed, recommending a thrusting technique as an alternative. Thrusting near the first rib, however, requires advanced training to perform effectively [7]. The protocol presented here offers a repeatable and versatile approach that may be advantageous when symptom relief is not achieved with counterstrain.

Work from Sucher utilized a combination of both OMT and ultrasound to target the pectoralis minor for alleviation of TOS symptoms [9]. Sucher's study accurately targeted the pectoralis minor muscle using ultrasound guidance for his OMT treatment of neuromuscular TOS. His treatment successfully employed a myofascial release technique to alleviate the dysfunction, with immediate resolution of symptoms. The protocol that we propose also utilizes myofascial release; however, we cannot judge the accuracy of our monitoring hand to the degree that Sucher was able to achieve.

Evidence suggests that OMT is a viable treatment option for patients with anatomical variations. In a study by Herring et al., the authors proposed myofascial release with respiratory assistance for treating TOS caused by a cervical rib [10]. The patient in that case presented with symptoms similar to ours, including the pain radiating down the ulnar aspect of the arm. After treatment, the patient reported immediate alleviation of symptoms, and underwent multiple treatments, with each session delaying the onset of symptoms [10]. This supports the potential viability of the proposed protocol for treating individuals with anatomical variations, such as a cervical rib. 

Normally, the first rib is elevated in cases of TOS [11]. This elevation typically causes compression of the subclavian artery and, potentially, the lower trunk of the brachial plexus [11]. However, in our patient, the first rib was depressed, making this a unique presentation. A depressed first rib suggests a possible myofascial mechanism underlying the symptoms.

The variability in osteopathic skill sets presents a challenge, but the techniques used in this protocol are foundational and a part of the standard curriculum in osteopathic training, ensuring replicability among practitioners. This broad accessibility makes the proposed approach a practical resource for resolving TOS in diverse clinical settings. Additionally, some clinics may not have an access to ultrasound. Although not as precise as the ultrasound-guided model, this approach ensures that practitioners without advanced equipment can still effectively treat TOS.

## Conclusions

The approach that was put forward offers a replicable treatment protocol for neurogenic TOS that can be utilized by those who practice osteopathic manipulation. This approach allows us to target multiple musculoskeletal dysfunctions within the thoracic outlet. Our patient was unique since he did not have an elevated first rib, confirming the versatility of the proposed protocol. Additionally, it provides an alternative to the traditional counterstrain model as well as the ultrasound-guided approach. Though there are multiple ways to treat this dysfunction, we believe that our article may serve as an easily replicable approach, which can be utilized by clinics with fewer resources. Though we cannot validate the replicability of results due to the small sample size, the techniques themselves are taught within the osteopathic curriculum within the first two years. 

In the future, we would like to explore the long-term efficacy of this protocol and compare it with other proposed treatment modalities. It would also be helpful to assess the application of this technique in a variety of clinical settings and by multiple osteopathic practitioners with varying levels of expertise. Studies analyzing the protocol’s effectiveness across diverse populations, as well as its integration with other approaches such as physical therapy, would provide more insights into its overall efficacy. We would like to integrate the proposed protocol in a population with regular follow-up to assess the long-term efficacy of this treatment. 
